# Supplementary material for: Assessing the impact of lateral flow testing strategies on within-school SARS-CoV-2 transmission and absences: A modelling study
Source: PLoS Comput Biol. 2022 May 27;18(5):e1010158. doi: 10.1371/journal.pcbi.1010158 (PMC9182264; doi:10.1371/journal.pcbi.1010158)
Supplement: S1 Text — Description of model algorithm and assumptions in detail. Descriptions of infectiousness over time, the parameterisation of within-school transmission, external infection, and recovery and immunity, extending the model to include interaction between year groups, and test probability profiles for symptomatic and asymptomatic individuals. (PDF) [file pcbi.1010158.s001.pdf]

Supporting Information. Assessing the impact of lateral flow testing strategies on within-school SARS-CoV-2 transmission and absences: a modelling study

Trystan Leng[1,2*], Edward M. Hill[1,2], Robin N. Thompson[1,2], Michael J. Tildesley[1,2], Matt J. Keeling[1,2], Louise Dyson[1,2]

1. The Zeeman Institute for Systems Biology & Infectious Disease Epidemiology Research, School of Life Sciences and Mathematics Institute, University of Warwick, Coventry, United Kingdom

2. JUNIPER – Joint UNIversities Pandemic and Epidemiological Research, https://maths.org/juniper/

S1 Text: Supporting methods

Simulation model

In this paper, we consider a stochastic individual-based model with a daily time-step of a secondary school comprising of five year groups of 200 pupils. Epidemics are simulated for one week prior to schools opening, and then over a seven week half-term. This supporting text outlines the steps within the model algorithm.

Prior to the model simulation, we assign the infectious status of each pupil, with the number of pupils who are susceptible, infected, and immune to infection drawn from a multinomial distribution with parameters $n = 1000, p = [1 - I_{init} - R_{init}, I_{init}, R_{init}]$. For each initially infected individual we assigned a random number of days since infection, between 1 and 15 days. We also assigned whether pupils would be symptomatic if they became infected. For such individuals, we sampled the number of days until symptom onset after becoming infected from a Gamma distribution, with shape 5.62 and scale 0.98 [1], considering only integer values. Initially infected symptomatic individuals who display symptoms prior to the beginning of the simulation are determined, assumed to have taken a PCR test on the day of symptom onset, with the PCR test result determining whether those individuals are initially isolated.

Throughout the simulation, schools implement one of six control strategies: (i) isolation of year group bubbles; (ii) twice weekly mass testing and isolation of year group bubbles; (iii) serial contact testing; (iv) twice weekly mass testing and serial contact testing; (v) twice weekly mass testing; or (vi) No school-level testing or isolation of year group bubbles. After the beginning of the model simulation, each day progresses in five stages, with the specific action at each step dependent on the implemented school control strategy. Specifically, on a given day d:

1. **Symptomatic pupils seek a PCR test.** Symptomatic pupils seek a PCR test on their day of symptom onset. Pupils that test positive do not attend school and isolate for the next ten days from the day after symptom onset. If the school term has started, if their year group bubble is not already isolating, and if the school is employing strategies (i) or (ii), the year group bubble of the pupil testing positive is assigned to isolate from the day after the return of the PCR result until 10 days after the identification of the positive PCR result. If the school term has started, and if the school is employing strategies (iii) or (iv), the year group bubble of the pupil testing positive is assigned to take an LFT from the day after the return of the PCR result until 7 days after the identification of the positive case.

2. **Scheduled pupils attending school take an LFT (only strategies ((ii)-(v)).** If d is a school day, all pupils scheduled to take an LFT who are not isolating take a test. If a pupil tests positive, they do not attend school, and seek a confirmatory PCR test. We assume that true infected pupils who return a positive LFT result also return a positive PCR result. If the school term has started, if their year group bubble is not already isolating, and if the school is employing strategy (ii), the year group bubble of the pupil testing positive is assigned to isolate from the next day for a number of days dependent on the outcome of the confirmatory PCR test. If the school term has started, and if

the school is employing strategies (iii) or (iv), the year group bubble of the pupil testing positive is assigned to take an LFT from the next day for a number of days dependent on the outcome of the confirmatory PCR test.

3. **Within-school transmissions occur.** If d is a school day, infected individuals i who are not isolating infect susceptible pupils j within their year group bubble who attend school that day with probability $\tau(i, j)$. Day d is set as the day of infection for newly infected individuals, and the number of days since infection is now recorded. If the pupil was assigned to be symptomatic if infected, the day they will develop symptoms is recorded.

4. **External transmissions occur.** Susceptible individuals who are not isolating are infected via external transmission with probability ϵ. Day d is set as the day of infection for newly infected individuals, and the number of days since infection is now recorded, and if they are symptomatic, the day they will develop symptoms is recorded.

5. **Updates to the number of days since infection of previously infected pupils.** Before the beginning of the next day, the number of days since infection for all individuals infected before day d is increased by 1. Based on the increased day since infection value, we update the probability of testing positive to a PCR or an LFT, as well as their infectivity $\Gamma_I(d - d_0)$.

To improve the computational speed of the simulation, stages 3 and 4 can be combined. The probability of infection to each susceptible individual j who is not isolating can be calculated as $1-((1-\epsilon)\times\prod_i(1-\tau(i, j)))$. However, we delineate the two stages to compare different strategies using the same set of random numbers for the transmission events on each day for each of the six strategies.

Infectiousness over time

In our model, infected pupils attending school (i.e. those not isolating and it being a school day) transmit infection to other pupils within their year group with a probability dependent on the time elapsed since their infection. Specifically, we assume that the relative probability of transmission since the day of infection is given by a Gamma distribution with shape 5.62 and scale 0.98 [2]. As the simulation uses a daily timestep, these probabilities fall on the integer values of this infectiousness distribution. Furthermore, this infectivity profile distribution was derived from data from known source-recipient pairs [3], with an assumed incubation period distribution (Gamma distributed with shape 5.807 and scale 0.948 [1]), under the assumption that the generation time and incubation period are independent. When sampling the number of days until symptom onset, we considered a discretised version of this distribution, considering symptom onset to occur a discrete number of days since infection. The discretised versions of both the infectivity profile and the incubation period profile are visualised in Figure A. Fifteen days after becoming infected, we assume that pupils are no longer infectious, and remain immune to infection for the rest of the simulation.

Parameterising within-school transmission

We defined the level of transmission within a school by a parameter, K. Specifically, K defined the expected number of secondary cases from an infected symptomatic pupil, assuming a fully susceptible school population, that the impact of depletion of susceptibles is negligible, and that the symptomatic pupil attends school each day of their infectious period. In reality, due to the small size of school populations, the depletion of susceptible individuals is never negligible. Further, symptomatic pupils will isolate after they test positive to either a PCR test or an LFT, and will not attend school at weekends. Hence an infected symptomatic pupil would be expected to infect less than K other pupils. The appropriate choice of K is unclear, and is likely to be influenced by a variety of factors that may vary from school to school, including the success of other within-school social distancing measures and the epidemiological characteristics of the dominant strain of SARS-CoV-2 in circulation in the local area. Due to this uncertainty, we considered a range of different values of K, specifically, $K \sim Unif(1, 5)$. We assumed that asymptomatic individuals were between 30 and 70% as infectious as those that develop symptoms, i.e. if a symptomatic pupil was expected to infect K other pupils over the course of their infection, an asymptomatic pupil was expected to infect $0.3 - 0.7 \times K$ other pupils.

External infection

As well as transmission within school, we assumed that all pupils who were not isolating had a constant probability of external infection each time step. Denoted ϵ, this parameter represents the daily probability of infection to pupils from the wider community. In our baseline parameterisation, we set ϵ such that an

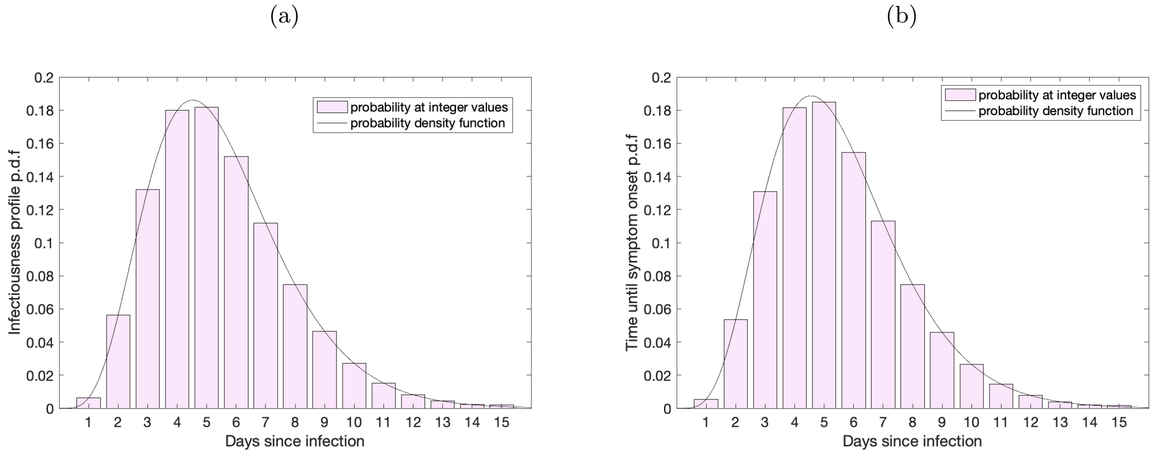

Figure A: **Discretised infectivity profile and incubation period distributions.** Here we plot (a) the infectivity profile, and (b) the incubation period distributions used in this manuscript. Black curves show the continuous probability density functions. Pink bars show the probabilities obtained by considering a discretised distribution that progresses in time steps of days. We used a previously defined infectivity profile distribution[2] derived in reference to an assumed incubation period distribution[1], under the assumption that the generation time and incubation period are independent.

average of 10% of pupils became infected by the end of the half-term under an isolation of year groups policy. When isolating, we assumed that individuals adhered and effectively isolated, meaning they had no probability of becoming infected whilst isolating.

Interaction between year groups

While our main analyses considers a scenario where year group bubbles are effective and exclusive (i.e. there is no transmission between year groups), the impact of interaction between year groups can be incorporated into our model and is considered in our sensitivity analysis (see S2 Text). We let $\lambda_i(d)$ denote the expected number of secondary infections from a symptomatic infected individual i on day d, assuming all their contacts are susceptible, and let N_k denote the number of individuals in year group k. $\lambda_i(d)$ is then given by:

$$\lambda_i(d) = \sum_{j \in \{\text{same year}\}} \tau(i,j) = \sum_{j \in \{\text{same year}\}} \frac{K \times \Gamma_I(d-d_0)}{N_1 - 1} = K \times \Gamma_I(d-d_0) \tag{1}$$

When considering interactions between year group bubbles, we assume frequency-dependent transmission, i.e. the expected number of secondary infections from an infected individual remains constant. Assuming there is complete random mixing between year groups, $\lambda_i(d)$ satisfies:

$$\lambda_i(d) = \sum_{j \in \{\text{school}\}} \tau^*(i,j), \text{ where } \tau^*(i,j) = \frac{K \times \Gamma_I(d-d_0)}{(\sum_{k=1}^{5} N_k) - 1} \tag{2}$$

The impact of interaction between year groups is captured by the parameter α, defined such that for $\alpha = 0$, Equation (1) is satisfied, and for $\alpha = 1$, Equation (2) is satisfied. Specifically, we define the probability of infection between a symptomatic infected individual i in year group i to a susceptible individual j in any year as:

$$\tau^*(i,j) = \frac{K \times \Gamma_I(d-d_0)}{(N_1 - 1) + \alpha \sum_{k=2}^{5} N_k} \times \begin{cases} 1, \text{ if } i \text{ and } j \text{ are in the same year and both attend school} \\ \alpha, \text{ if } i \text{ and } j \text{ are in different years and both attend school} \\ 0, \text{ if } i \text{ or } j \text{ does not attend school} \end{cases} \tag{3}$$

To obtain expressions for an infected asymptomatic individual, the above expressions are multiplied by the factor a, which denotes the relative infectiousness of asymptomatic individuals.

Test probability profiles for symptomatic and asymptomatic individuals

While the probability of testing positive through time has been detailed for both PCR and LFT tests, prior analyses have typically been based on symptomatic individuals[4]. The probability of testing positive is likely a function of viral load; while symptomatic and asymptomatic individuals have similar average peak viral loads and proliferation stage durations, their average duration of clearance stages has been observed to differ[5,6]. In this paper, we assumed that the probability of asymptomatic individuals testing positive to both PCR and LFT was equal to that of symptomatic individuals until the peak of infection, but then decays more rapidly, such that the probability of an asymptomatic individual testing positive at 6.7 days after the peak should equal the probability of a symptomatic individual testing positive at 10.5 days after the peak (corresponding with estimates that the average duration of clearance of 10.5 days is symptomatic cases and 6.7 days in asymptomatic cases[5]). We applied the same method to the 95% credible intervals provided by[4] to obtain high and low sensitivity test probability profiles. However, we express that this is an area of considerable uncertainty. Future studies detailing the testing probability of asymptomatic individuals, and the specific relationship between viral load and testing probability, would be a valuable contribution to this area.

(a) (b)

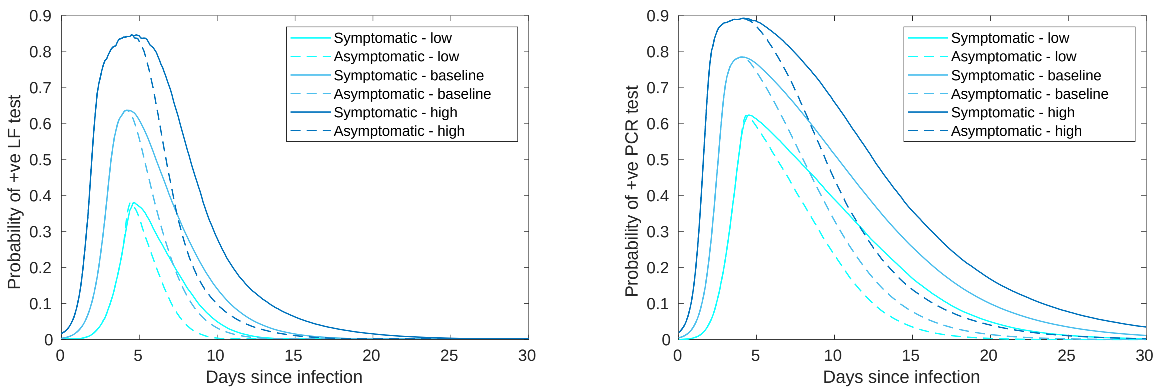

Figure B: **Probabilities of testing positive through time for symptomatic and asymptomatic individuals.** We assumed that the probability of positive **(a)** LFTs, and **(b)** PCR tests in symptomatic and asymptomatic individuals were equal during the proliferation stage of the virus, but that the probability of asymptomatic individuals testing positive decayed faster in the clearance stage, owing to a shorter mean clearance duration of 6.7 days[5]. We took the above profiles for symptomatic individuals directly from[4]

.

As a final step, we adjusted for the specificity of LFTs by setting the minimum daily probability of testing positive to be 0.03%[7].

References

[1] Lauer SA, Grantz KH, Bi Q, Jones FK, Zheng Q, Meredith HR, et al. The Incubation Period of Coronavirus Disease 2019 (COVID-19) From Publicly Reported Confirmed Cases: Estimation and Application. Annals of Internal Medicine. 2020 3.

[2] Hart WS, Maini PK, Thompson RN. High infectiousness immediately before COVID-19 symptom onset highlights the importance of continued contact tracing. Elife. 2021;10:e65534.

[3] Ferretti L, Ledda A, Wymant C, Zhao L, Ledda V, Abeler- Dorner L, et al. The timing of COVID-19 transmission. medRxiv. 2020:2020.09.04.20188516. Available from: http://medrxiv.org/content/early/2020/09/16/2020.09.04.20188516.abstract.

[4] Hellewell J, Russell TW, Beale R, Kelly G, Houlihan C, Nastouli E, et al. Estimating the effectiveness of routine asymptomatic PCR testing at different frequencies for the detection of SARS-CoV-2 infections. BMC Medicine. 2021;19:106. Available from: https://bmcmedicine.biomedcentral.com/articles/10.1186/s12916-021-01982-x.

[5] Kissler SM, Fauver JR, Mack C, Olesen SW, Tai C, Shiue KY, et al. Viral dynamics of acute SARS-CoV-2 infection and applications to diagnostic and public health strategies. PLoS biology. 2021;19(7):e3001333.

[6] Uhm JS, Ahn JY, Hyun J, Sohn Y, Kim JH, Jeong SJ, et al. Patterns of viral clearance in the natural course of asymptomatic COVID-19: Comparison with symptomatic non-severe COVID-19. Int J Infect Dis. 2020 oct;99:279-85.

[7] Joint PHE Porton Down & University of Oxford SARS-CoV-2 test development and validation cell. Preliminary report from the Joint PHE Porton Down & University of Oxford SARS-CoV-2 test development and validation cell: Rapid evaluation of Lateral Flow Viral Antigen detection devices (LFDs) for mass community testing; 2020. Available from: https://www.ox.ac.uk/sites/files/oxford/media_wysiwyg/UK%20evaluation_PHE%20Porton%20Down%20%20University%20of%20Oxford_final.pdf.
