# Supplementary material for: Assessing the impact of lateral flow testing strategies on within-school SARS-CoV-2 transmission and absences: A modelling study
Source: PLoS Comput Biol. 2022 May 27;18(5):e1010158. doi: 10.1371/journal.pcbi.1010158 (PMC9182264; doi:10.1371/journal.pcbi.1010158)
Supplement: S2 Text — Description of the univariate sensitivity analysis performed, and the results from this analysis. (PDF) [file pcbi.1010158.s002.pdf]

Supporting Information. Assessing the impact of lateral flow testing strategies on within-school SARS-CoV-2 transmission and absences: a modelling study

Trystan Leng[1,2*], Edward M. Hill[1,2], Robin N. Thompson[1,2], Michael J. Tildesley[1,2], Matt J. Keeling[1,2], Louise Dyson[1,2]

1. The Zeeman Institute for Systems Biology & Infectious Disease Epidemiology Research, School of Life Sciences and Mathematics Institute, University of Warwick, Coventry, United Kingdom

2. JUNIPER – Joint UNIversities Pandemic and Epidemiological Research, https://maths.org/juniper/

S2 Text: Sensitivity analysis

There is considerable uncertainty surrounding many of the parametric assumptions that underpin the model. Accordingly, we performed a univariate sensitivity analysis to understand the impact these assumptions have on our findings (Figures A to D). We performed the sensitivity analysis using a one-at-a-time procedure, with one parameter varied from its baseline value whilst all other parameters were kept constant. For each parameter considered we defined a lower than baseline parameterisation and higher than baseline parameterisation. Where parameters were drawn from a distribution for the main analysis, we took the midpoint value of that parameter as the baseline value for the sensitivity analysis (Table 1 of the main text).

In Figure A, we consider the impact of parametric assumptions on the infections, absences, and number of LFTs taken by the end of the half-term under school reopening strategies (i)-(v). For all strategies, either the level of within-school transmission or the probability of infection from the community had the highest impact on infections over the half-term. For strategies involving lateral flow testing, the sensitivity of LFTs also had a moderate impact on transmission. Probability of community infection could also have a large impact on the number of school days missed per pupil, as well as the number of LFTs taken per pupil. Across the range of parametric assumptions considered, absences remained at very low levels for strategies (iii)-(v), i.e. those strategies not involving the isolation of year groups. For strategies (iii) and (iv), assumptions that led to higher (lower) levels of infection typically led to a higher (lower) numbers of tests being taken, with the exception of PCR sensitivity and the percentage of pupils who develop symptoms. For strategy (v), where all pupils take an LFT twice a week if they attend school, the number of LFTs taken per pupil is relatively unaffected by the underlying parametric assumptions. For all strategies, assuming that LFT or PCR sensitivity is higher than our baseline assumption reduces infections over the half-term, while assuming levels of within-school transmission, community transmission, or the relative infectiousness of asymptomatics are higher than our baseline assumption increases infections over the half-term. These parameters impact within-school transmission in opposing directions, meaning their inference may be challenging even with empirical data.

We next compare the ordering in terms of the relative effectiveness of strategies under each parametric assumption, with regards to average infections, average absences, and average testing demand in turn. Across the range of assumptions considered, a strategy of twice weekly mass testing combined with isolation of year groups bubble was the most effective strategy at reducing the expected number of infections over the half-term, followed by twice weekly mass testing combined with serial contact testing, while serial contact testing alone was the least effective strategy (Figure B). While twice weekly mass testing was more effective than the isolation of year group bubbles for the majority of assumptions considered, an isolation of year group bubbles strategy had fewer infections by the end of the half-term under a number of the considered assumptions. Twice weekly mass testing relies solely upon the detection of cases to control infections, hence this strategy became comparably less effective when the sensitivity of LFTs was assumed to be low. This strategy was also less effective when the level of within-school transmission was lower. When transmission within-school is low, the benefits of detecting cases is marginal; in contrast, strategies involving isolation still reduce transmission by the requirement to isolate, limiting their probability of community infection.

Twice weekly mass testing also became less effective at higher levels of community infection, indicating that such a strategy may not be appropriate by itself when prevalence is high in the community. A strategy of isolating year groups was also marginally more effective when a higher proportion of infected pupils developed symptoms, and when there was a higher level of interaction between year groups.

Considering absences, a strategy of twice weekly mass testing combined with isolation of year groups bubble resulted in the highest number of absences over the half-term, followed by the isolation of year groups alone (Figure C). Strategies that did not involve the isolation of year group bubbles consistently led to considerably fewer absences; serial contact testing typically led to the fewest absences, although the order of the strategies not involving isolation varied between parametric assumptions. Absence levels are a function of both prevalence and surveillance. For example, while serial contact testing typically led to fewer absences than when this measure was combined with twice weekly mass testing, in virtue of fewer tests being taken, the combined strategy typically led to fewer absences than a strategy of twice weekly mass testing alone, in virtue of lower levels of infections. The impact of higher levels of infections under some parametric assumptions was sufficient to change the ordering of strategies, such as at higher levels of within-school transmission.

Considering testing demand, a strategy of twice weekly mass testing combined with serial contact testing resulted in the highest mean number of LFTs taken per pupil, typically followed by a strategy of twice weekly mass testing alone (Figure D). By definition of the strategy, the isolation of year group bubbles required no LFTs. The higher numbers of infections impacted the number of LFTs required for a strategy of serial contact testing and a strategy of twice weekly mass testing combined with isolation of year group bubbles in opposite directions. For a strategy of serial contact testing, parametric assumptions that increased infections typically increased testing demand. In contrast, increased infections could decrease testing demand for a strategy combining twice weekly mass testing with the isolation of year group bubble, as a consequence of isolating pupils not being required to take LFTs. This effect was sometimes sufficient to change the ordering of these strategies, such as when a higher probability of infection from the community was assumed.

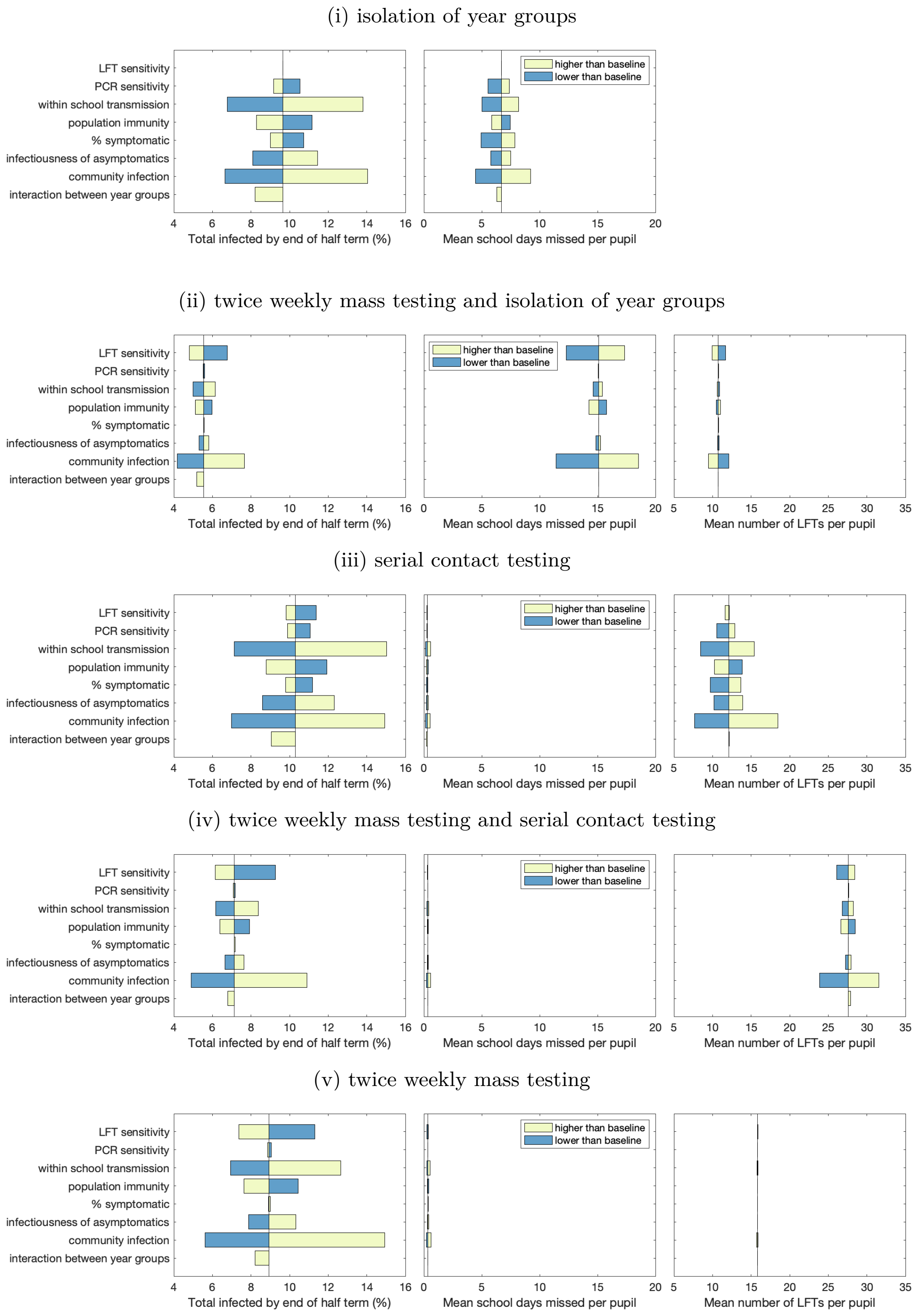

Figure A: **Univariate sensitivity analysis of school reopening strategies.** Sensitivity analyses considering the impact of model assumptions on the total number of infections by the end of half-term (left column), the mean school days missed per pupil (centre column), and the mean number of LFTs taken per pupil (right column), for the strategies (i) isolation of year groups, (ii) twice weekly mass tests and isolation of year groups, (iii) serial contact testing, (iv) twice weekly mass tests and serial contact testing, and (v) twice weekly mass tests. We consider the impact of varying our assumptions surrounding: LFT sensitivity, PCR sensitivity, the level of within-school transmission (K), the initial level of immunity among pupils (R_{init}), the percentage of infected pupils who develop symptoms, the relative infectiousness of asymptomatic pupils (a), the probability of external infection from the community (ϵ), and interaction between year groups (α). Higher than baseline and lower than baseline parameter choices are outlined in Table 1 in the main text. Results produced from 2,000 simulations for each parametric assumption, with light (dark) bars depicting outcomes using the higher (lower) than baseline parameter values.

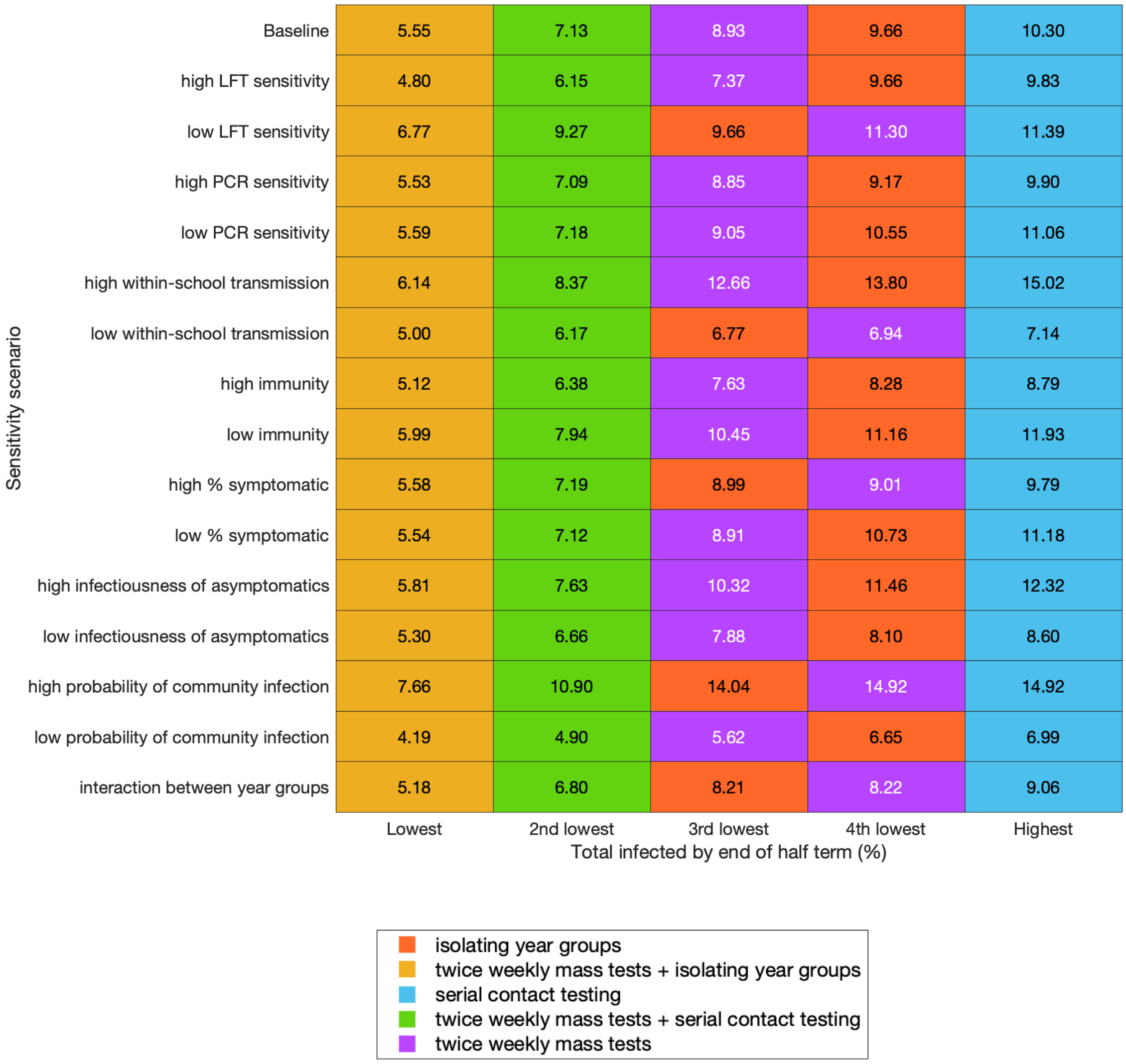

Figure B: **The relative impact of school reopening strategies on transmission from univariate sensitivity analysis.** Results from 2000 simulations under a different parametric assumption considered in the univariate sensitivity analysis. Each row displays the mean percentage of pupils infected by the end of the half-term, ordered from the strategy resulting in the lowest number of infections (left) to the strategy with the highest number of infections (right). Cells are coloured according to the strategy they correspond to. The following strategies are considered: isolation of year group bubbles (orange), twice weekly mass tests and isolation of year groups (yellow), serial contact testing (blue), twice weekly mass tests and serial contact testing (green), and twice weekly mass tests (purple).Parametric choices considered in the sensitivity analysis that are higher and lower than the baseline parameter choices are outlined in Table 1 in the main text.

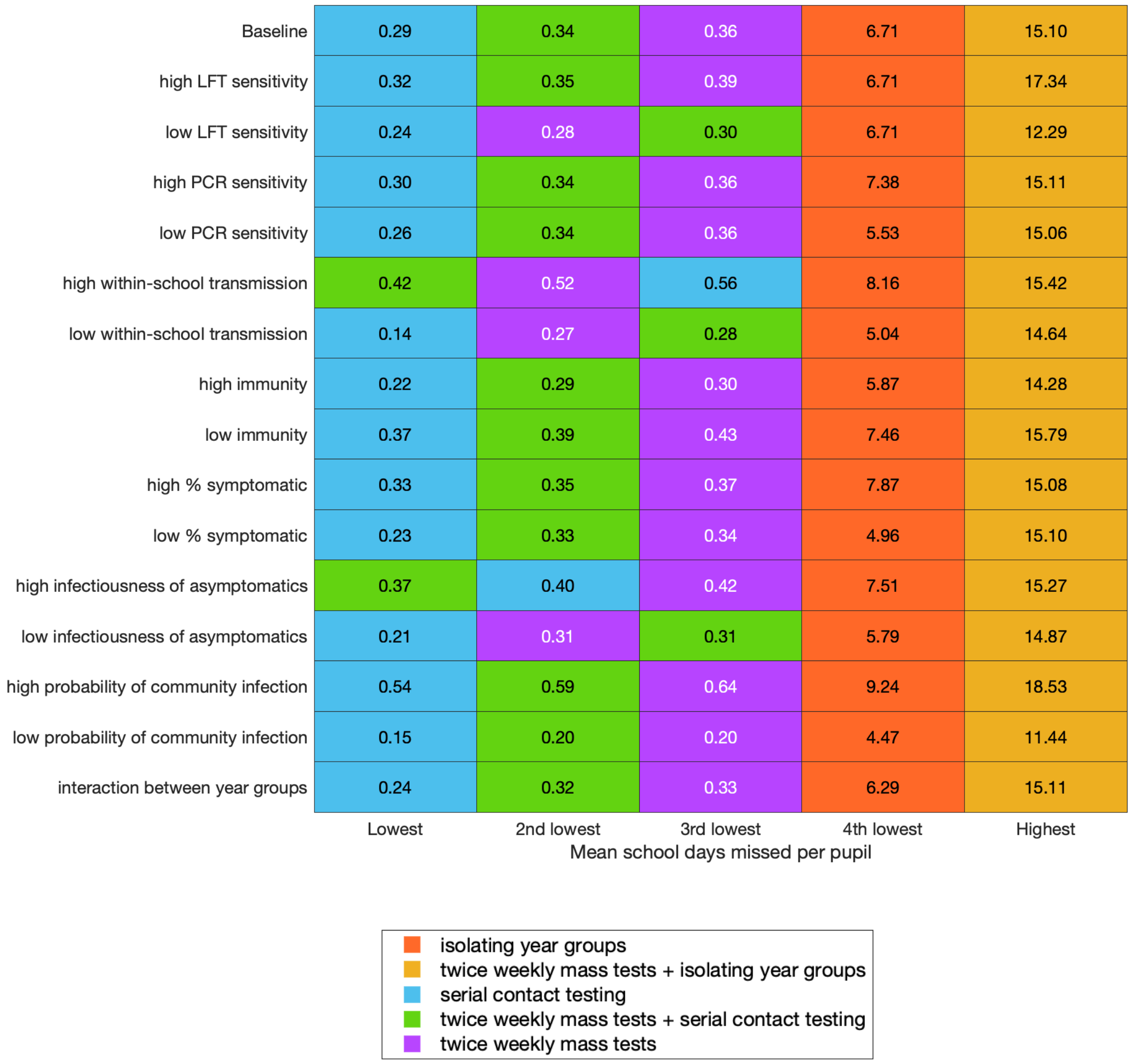

Figure C: **The relative impact of school reopening strategies on absences from univariate sensitivity analysis.** Results from 2000 simulations under a different parametric assumption considered in the univariate sensitivity analysis. Each row displays the mean number of school days missed per pupil by the end of the half-term ordered from the strategy resulting in the lowest number of absences (left) to the strategy with the highest number of absences (right). Cells are coloured according to the strategy they correspond to. The following strategies are considered: isolation of year group bubbles (orange), twice weekly mass tests and isolation of year groups (yellow), serial contact testing (blue), twice weekly mass tests and serial contact testing (green), and twice weekly mass tests (purple). Parametric choices considered in the sensitivity analysis that are higher and lower than the baseline parameter choices are outlined in Table 1 in the main text.

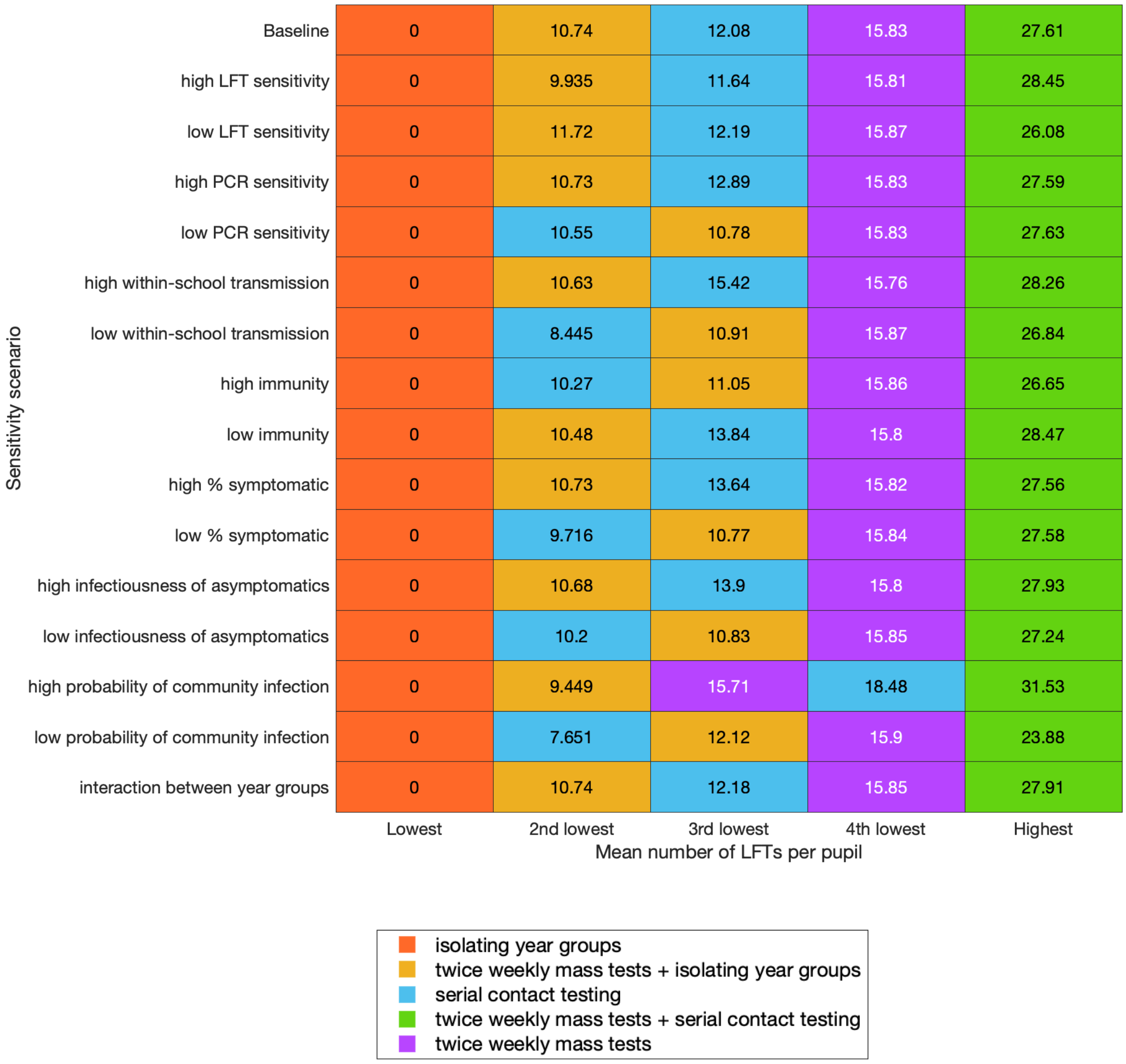

Figure D: **The relative impact of school reopening strategies on LFT demand from univariate sensitivity analysis.** Results from 2000 simulations under a different parametric assumption considered in the univariate sensitivity analysis. Each row displays the mean number of LFTs taken per pupil by the end of the half-term ordered from the strategy resulting in the lowest number of LFTs taken (left) to the strategy with the highest number of LFTs taken (right). Cells are coloured according to the strategy they correspond to. The following strategies are considered: isolation of year group bubbles (orange), twice weekly mass tests and isolation of year groups (yellow), serial contact testing (blue), twice weekly mass tests and serial contact testing (green), and twice weekly mass tests (purple). Parametric choices considered in the sensitivity analysis that are higher and lower than the baseline parameter choices are outlined in Table 1 in the main text.
