# Supplementary material for: Assessing the impact of lateral flow testing strategies on within-school SARS-CoV-2 transmission and absences: A modelling study
Source: PLoS Comput Biol. 2022 May 27;18(5):e1010158. doi: 10.1371/journal.pcbi.1010158 (PMC9182264; doi:10.1371/journal.pcbi.1010158)
Supplement: S3 Text — The impact of accounting for a probability of transmission between pupils on days in which they test positive, is explored. (PDF) [file pcbi.1010158.s003.pdf]

Supporting Information. Assessing the impact of lateral flow testing strategies on within-school SARS-CoV-2 transmission and absences: a modelling study

Trystan Leng[1,2*], Edward M. Hill[1,2], Robin N. Thompson[1,2], Michael J. Tildesley[1,2], Matt J. Keeling[1,2], Louise Dyson[1,2]

1. The Zeeman Institute for Systems Biology & Infectious Disease Epidemiology Research, School of Life Sciences and Mathematics Institute, University of Warwick, Coventry, United Kingdom

2. JUNIPER – Joint UNIversities Pandemic and Epidemiological Research, https://maths.org/juniper/

S3 Text: Transmission on test days

In our main analysis, we assumed that LFTs were taken before the pupil attended school each day. Consequently, pupils that tested positive did not attend school (with length of isolation dependent upon the result of a PCR confirmatory test) and had no risk of transmitting infection to other pupils. However, we could also consider a situation where LFTs are taken within school, with an associated risk of pupils testing positive transmitting infection before detection.

We consider here the impact of transmission on test days, considering the extreme example where pupils who test positive to an LFT only start isolating from the day after detection. For all strategies considered, a similar but slightly higher mean number of infections occurred assuming a risk of transmission on test days versus no risk of transmission on test days, with larger outbreaks also occurring more frequently. The largest increase in infections occurred for a strategy of twice weekly mass testing; 9.4% (95% prediction interval (PI): 6.0-16.3%) of pupils were infected by the end of the half-term assuming no risk of transmission on test days, increasing to 11.0% (95% PI: 6.3-22.0%) assuming a risk of transmission on test days.

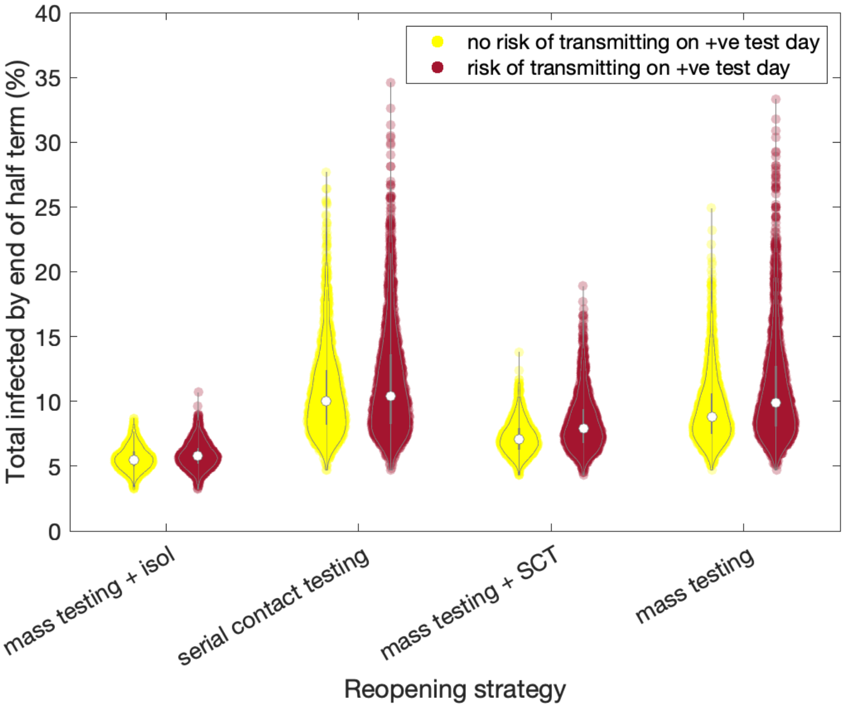

Figure A: **Exploring the impact of transmission on day of detection through lateral flow tests.** For all strategies involving lateral flow testing, we display violin plots of the total number of pupils infected by the end of the half-term assuming there is no risk of detected individuals transmitting on the day they tested positive (yellow) and assuming detected pupils do not isolate until the day after detection (burgundy). The strategies displayed are: twice weekly mass testing and isolating year groups (far left), serial contact testing (middle left), twice weekly mass testing and serial contact testing (middle right), and twice weekly mass testing alone (far right). Results produced from 2,000 simulations. In all violin plots, the circle marker denotes the median and the black bars the 50% prediction intervals.
