# Supplementary material for: Assessing the impact of lateral flow testing strategies on within-school SARS-CoV-2 transmission and absences: A modelling study
Source: PLoS Comput Biol. 2022 May 27;18(5):e1010158. doi: 10.1371/journal.pcbi.1010158 (PMC9182264; doi:10.1371/journal.pcbi.1010158)
Supplement: S4 Text — The impact of higher levels of immunity among pupils on the effectiveness different reopening strategies is explored. (PDF) [file pcbi.1010158.s004.pdf]

Supporting Information. Assessing the impact of lateral flow testing strategies on within-school SARS-CoV-2 transmission and absences: a modelling study

Trystan Leng[1,2*], Edward M. Hill[1,2], Robin N. Thompson[1,2], Michael J. Tildesley[1,2], Matt J. Keeling[1,2], Louise Dyson[1,2]

1. The Zeeman Institute for Systems Biology & Infectious Disease Epidemiology Research, School of Life Sciences and Mathematics Institute, University of Warwick, Coventry, United Kingdom

2. JUNIPER – Joint UNIversities Pandemic and Epidemiological Research, https://maths.org/juniper/

S4 Text: Increasing levels of immunity

In the main text, for the level of immunity among pupils prior to schools reopening we assumed $R_{init} = 20\%$, reflecting observed levels of immunity among the general population in England in January 2021 [1]. In our sensitivity analysis we considered $R_{init} = 10\%$ and $R_{init} = 30\%$ (S2 Text) chosen to reflect uncertainty in immunity levels at that period in time.

However, levels of immunity amongst secondary school aged children at later dates may be considerably higher than this, either because of natural immunity or because of vaccination. Thus, in this section we consider a wide range of initial levels of immunity (varying R_{init} from 0% to 90%), and consider the relative impact of different reopening strategies. All other parameters remain the same as in the main paper. The year group of each initially immune pupil is selected randomly. In reality, if immunity in the population is the result of a within-school epidemic, there could be heterogeneity in prior immunity between year groups. Nevertheless, by varying R_{init} we can gain a qualitative insight into the impact of existing levels of immunity on epidemiological trends.

(a) (b)

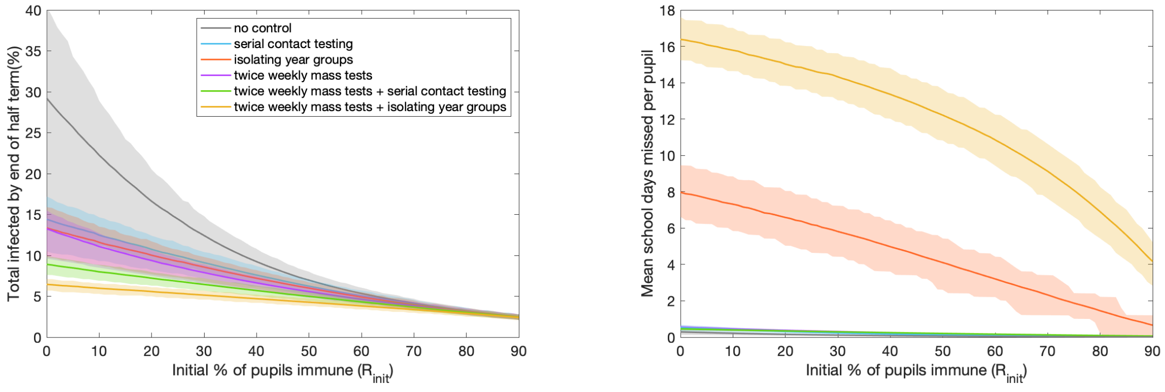

Figure A: **Exploring the impact of initial levels of immunity amongst pupils.** We study the impact of varying the initial level of immunity amongst pupils R_{init} (from 0% to 90% in 1% increments) on **(a)** the total number of pupils infected by the end of the half-term, and **(b)** the mean school days missed per pupil within a school over the course of the half-term. Solid line traces correspond to the mean value attained from 2,000 simulations. Shaded envelopes represent the 50% prediction intervals (these regions contain 50% of all simulations at each timepoint). The strategies displayed are: no control (grey), twice weekly mass testing (purple), serial contact testing (blue), isolating year group bubbles strategy (orange), twice weekly mass testing (purple), combined twice weekly mass testing and serial contact testing (green), and combined twice weekly mass testing and isolation of year group bubbles (yellow).

Across our considered range of R_{init} values, we retain the same strategy ordering as in the main paper in terms of their effectiveness at reducing transmission. As one would expect, higher levels of immunity result in fewer pupils being infected by the end of the half-term. Most starkly, with $R_{init} = 90\%$ there is only a marginal difference in the expected number of infections between strategies by the end of the half-term, with 2.5% (95% PI: 1.5%-3.5%) of pupils infected by the end of half-term with no control measures and 2.4% (95% PI: 1.5%-3.4%) infected by the end of half-term with a combined strategy of twice weekly mass testing and serial contact testing. Absences decrease as initial levels of immunity increase, although strategies involving the isolation of year group bubbles still cause considerable levels of absences at high levels of initial immunity. For example, for a strategy combining twice weekly mass testing with isolation and $R_{init} = 90\%$, the average pupil missed over 4 days of school.

References

[1] Office for National Statistics. Coronavirus (COVID-19) Infection Survey: antibody data for the UK, January 2021; 2021. Available from: https://www.ons.gov.uk/peoplepopulationandcommunity/healthandsocialcare/conditionsanddiseases/articles/coronaviruscovid19infectionsinthecommunityinengland/antibodydatafortheukjanuary2021.
