# Supplementary material for: Assessing the impact of lateral flow testing strategies on within-school SARS-CoV-2 transmission and absences: A modelling study
Source: PLoS Comput Biol. 2022 May 27;18(5):e1010158. doi: 10.1371/journal.pcbi.1010158 (PMC9182264; doi:10.1371/journal.pcbi.1010158)
Supplement: S5 Text — The impact of higher levels of within-school transmission on the effectiveness different reopening strategies is explored. (PDF) [file pcbi.1010158.s005.pdf]

Supporting Information. Assessing the impact of lateral flow testing strategies on within-school SARS-CoV-2 transmission and absences: a modelling study

Trystan Leng[1,2*], Edward M. Hill[1,2], Robin N. Thompson[1,2], Michael J. Tildesley[1,2], Matt J. Keeling[1,2], Louise Dyson[1,2]

1. The Zeeman Institute for Systems Biology & Infectious Disease Epidemiology Research, School of Life Sciences and Mathematics Institute, University of Warwick, Coventry, United Kingdom

2. JUNIPER – Joint UNIversities Pandemic and Epidemiological Research, https://maths.org/juniper/

S5 Text: Increasing within-school transmission

In this section, we consider the specific impact of increasing the transmissibility of SARS-CoV-2 within the school environment. The emergence of a series of more transmissible variants of the virus has considerably increased the reproduction number of SARS-CoV-2. Therefore, we consider here a yet wider range for the level of within school transmission (from $K = 0$ to $K = 10$, at increments of 0.1, whereas in the main text we used a narrower range of $K = 1$ to $K = 5$) to determine the effectiveness of school reopening strategies in the context of a more transmissible virus. We assume that all other parameters remain the same as in the main paper. In particular, we retain the same incubation profile, infectiousness profile, and test probability profile of SARS-CoV-2. We note that were new variants to alter these profiles, for example by shifting a higher proportion of infections to an individual's presymptomatic phase, these changes may have an impact on results.

(a)

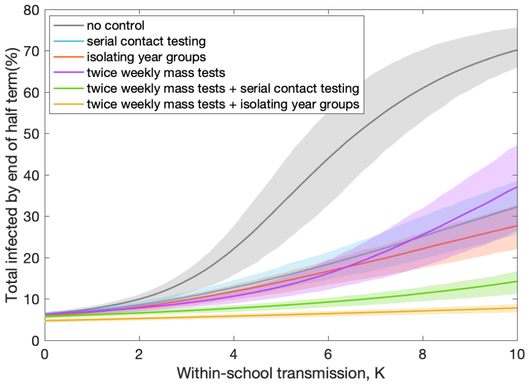

(b)

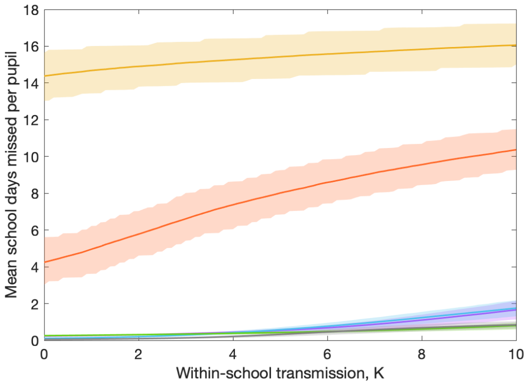

Figure A: **Exploring the impact of increasing within-school transmission.** We assess the impact of varying the baseline within-school transmission parameter K from 0 to 10 on **(a)** the total number of pupils infected by the end of the half-term, and **(b)** the mean school days missed per pupil within a school over the course of the half-term. We undertook simulations for values of K varying from 0 to 10 in increments of 0.1. Solid line traces correspond to the mean value attained from 2,000 simulations. Shaded envelopes represent the 50% prediction intervals (these regions contain 50% of all simulations at each value of K). The strategies displayed are: no control (grey), twice weekly mass testing (purple), serial contact testing (blue), isolating year group bubbles strategy (orange), twice weekly mass testing (purple), combined twice weekly mass testing and serial contact testing (green), and combined twice weekly mass testing and isolation of year group bubbles (yellow).

Across the range of values of K considered, the ordering of most strategies in terms of their effectiveness at

reducing transmission remains unchanged: serial contact testing is less effective than isolating year group bubbles, which is less effective than twice weekly mass testing alongside serial contact testing, which is less effective than twice weekly mass testing alongside isolating year group bubbles (Figure Aa). The exception to this is twice weekly mass testing. For very low values of K this strategy was less effective than the isolation of year group bubbles. For example, if $K = 0$, i.e. there is no transmission within-schools, there is no benefit conferred from the detection of cases under a strategy of twice weekly mass testing. In contrast, there is still a minor benefit to the isolation of year group bubbles strategy, as isolating individuals are assumed not to be at risk of infection from the community. At intermediate values of K ($1.5 < K < 6.3$), twice weekly mass testing becomes more effective than either isolating year groups or serial contact testing. However, twice weekly mass testing alone lacks a mechanism to suppress local outbreaks, which becomes increasingly important for higher levels of transmission. Consequently, in the upper range of K values tested we find twice weekly mass testing again becomes less effective than either isolating year group bubbles or serial contact testing. Across the range of values of K considered, strategies combining twice weekly mass testing with either serial contact testing or isolating year group bubbles remained extremely effective at controlling transmission. At $K = 10$, the highest value considered, 70.2% (95% PI: 51.9%-78.9%) of pupils would be infected by the end of the half-term with no control measures; this is reduced to 14.2% (95% PI: 8.1%-24.0%) and 7.8% (95% PI: 5.2%-11.3%) for a strategy combining twice weekly mass tests with serial contact testing or isolating year groups, respectively. Increasing K increases the mean number of school days missed per pupil for all scenarios, though for all strategies not involving the isolation of year group bubbles absences remain relatively low.
