# Supplementary material for: Assessing the impact of lateral flow testing strategies on within-school SARS-CoV-2 transmission and absences: A modelling study
Source: PLoS Comput Biol. 2022 May 27;18(5):e1010158. doi: 10.1371/journal.pcbi.1010158 (PMC9182264; doi:10.1371/journal.pcbi.1010158)
Supplement: S6 Text — The impact of dividing year group into discrete non-interacting cohorts is on different reopening strategies is considered, exploring the assumptions of frequency and density dependent transmission in turn. (PDF) [file pcbi.1010158.s006.pdf]

Supporting Information. Assessing the impact of lateral flow testing strategies on within-school SARS-CoV-2 transmission and absences: a modelling study

Trystan Leng[1,2*], Edward M. Hill[1,2], Robin N. Thompson[1,2], Michael J. Tildesley[1,2], Matt J. Keeling[1,2], Louise Dyson[1,2]

1. The Zeeman Institute for Systems Biology & Infectious Disease Epidemiology Research, School of Life Sciences and Mathematics Institute, University of Warwick, Coventry, United Kingdom

2. JUNIPER – Joint UNIversities Pandemic and Epidemiological Research, https://maths.org/juniper/

S6 Text: The impact of cohorting

Our main analysis considers a situation where a school implements a bubbling policy at the level of year groups. Accordingly, we assume random mixing within year groups and no interaction between year groups. Here, we instead consider the impact of cohorting year groups into smaller units on the effectiveness of the six reopening strategies considered. We assume a bubbling policy at the level of cohorts, i.e. we assume random mixing within cohorts but no interaction between cohorts. Because of the exclusivity of cohorts, strategies involving isolation only isolate cohorts upon identification of a positive case, rather than the entire year. Similarly, strategies involving serial contact testing only test cohorts upon identification of a positive case.

Previous studies have considered the impact of reducing the number of pupils interacting within the school environment [1–3]. However, the impact of reducing the number of potential contacts pupils make within the school setting may depend upon the assumptions made regarding the nature of transmission within schools. If one assumes density dependent transmission, reducing the size of cohorts reduces the expected number of secondary infections from an infected individual. Alternatively, one could consider frequency dependent transmission, where reducing the size of cohorts does not reduce the expected number of secondary infections from an infected individual. We consider both of these assumptions in turn, for cohorts of sizes 25, 50, 100, and 200. Specifically, to consider density dependent transmission we set $N = 200$ in Equation 1 in the main text for all cohort sizes, while for frequency dependent transmission we set set $N = \{25, 50, 100, 200\}$.

Assuming density dependent transmission, cohorting is in and of itself very effective at reducing transmission (Figure Aa). Under a strategy of no additional controls, the expected number of infections by the end of the half-term for cohorts of 200 was 16.6% (95% PI: 6.8% - 42.5%), whereas for cohorts of size 25 we observe a lesser half-term incidence of 6.7% (95% PI 5.1 % - 8.5%). In fact, for cohorts of size 25, strategies involving either isolation or serial contact testing have only a limited impact - a strategy of twice weekly mass testing combined with isolating cohorts, with most stringent policy considered, reduces infections to 6.1% (95% PI 4.7 % - 7.6%). For most strategies considered, infections increase with cohort size, with the only exception being a strategy of twice weekly mass testing combined with isolating cohorts. At all cohort sizes, the order of the relative effectiveness of control strategies (i)-(v) is unchanged from the main analysis, with the ordering from least effective at reducing transmission to most effective as follows: (iii) serial contact testing, (i) isolation of year group bubbles, (v), twice weekly mass testing, (ii) twice weekly mass testing with isolation bubbles , (iv) twice weekly mass testing with serial contact testing. For all strategies, the mean school days missed per pupil increases with cohort size (Figure Ab).

If, instead, we assume frequency dependent transmission, cohorting alone is not an effective measure at reducing transmission (Figure Ba). While infections still increase with cohort size for a no control strategy or a strategy of twice weekly mass testing, this trend is reversed for strategies involving either isolation of cohorts of serial contact testing of cohorts. As isolation or serial contact testing is initiated after a positive case is identified, such strategies become more effective for larger group sizes, as there is the potential to avoid a greater number of transmission events after a positive case is detected. As for density dependent transmission the order of most effective control strategies is unchanged from the main analysis.

For strategies involving the isolation of cohorts, the mean number of school days missed per pupil increases considerably with cohort size, but for other strategies absence levels are relatively unaffected by cohort size (Figure Bb).

The effectiveness of cohorting at reducing transmission is sensitive to whether one assumes frequency dependent transmission or density dependent transmission within schools. However, under both assumptions, the ordering of effectiveness of different strategies is unchanged by cohort size. Therefore, while our results demonstrate the importance of understanding the nature of transmission within schools, they also demonstrate the robustness of our results regarding the relative effectiveness of different strategies at reducing transmission.

(a)

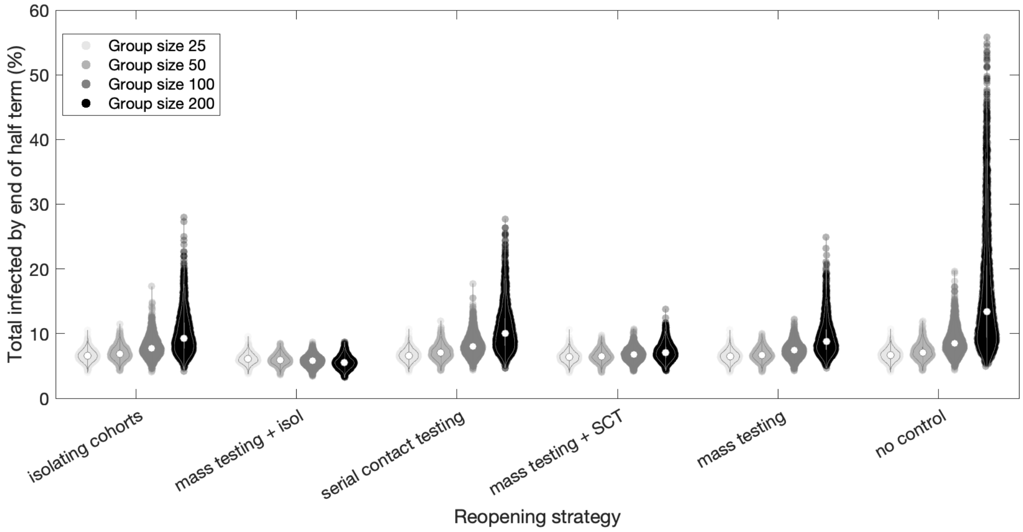

(b)

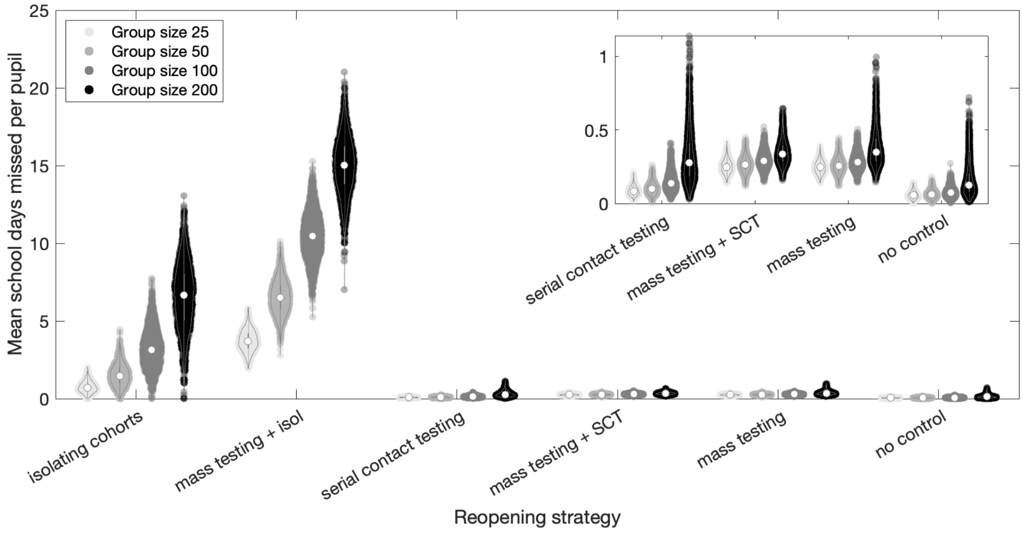

Figure A: **Exploring the impact of cohorting, assuming density dependent transmission.** Violin plots of (a) the total number of pupils infected by the end of the half-term and (b) the mean school days missed per pupil within a school over the course of a half-term, at cohorts of size: 25 (light grey), 50 (medium grey), 100 (dark grey), and 200 (black). We considered six different school reopening strategies (from left to right): (i) isolating cohorts, (ii) twice weekly mass testing and isolating cohorts, (iii) serial contact testing of cohorts, (iv) twice weekly mass testing and serial contact testing of cohorts, (v) twice weekly mass testing alone, and (iv) no control strategy. Results produced from 2,000 simulations. In all violin plots, the circle marker denotes the median and the black bars the 50% prediction intervals.

(a)

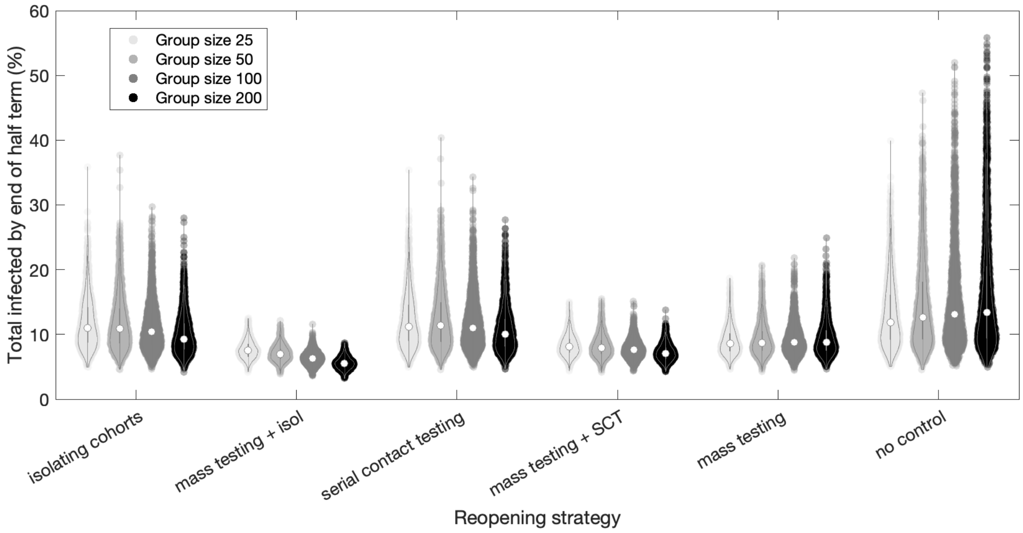

(b)

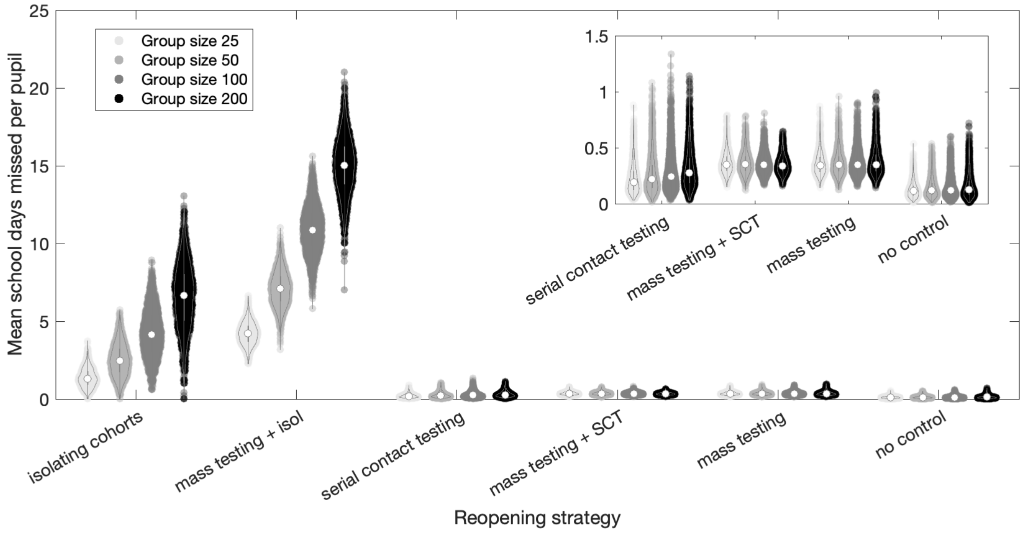

Figure B: **Exploring the impact of cohorting, assuming frequency dependent transmission.** Violin plots of (a) the total number of pupils infected by the end of the half-term and (b) the mean school days missed per pupil within a school over the course of a half-term, at cohorts of size: 25 (light grey), 50 (medium grey), 100 (dark grey), and 200 (black). We considered six different school reopening strategies (from left to right): (i) isolating cohorts, (ii) twice weekly mass testing and isolating cohorts, (iii) serial contact testing of cohorts, (iv) twice weekly mass testing and serial contact testing of cohorts, (v) twice weekly mass testing alone, and (iv) no control strategy. Results produced from 2,000 simulations. In all violin plots, the circle marker denotes the median and the black bars the 50% prediction intervals.

References

[1] Phillips B, Browne DT, Anand M, Bauch CT. Model-based projections for COVID-19 outbreak size and student-days lost to closure in Ontario childcare centres and primary schools. Scientific reports. 2021;11(1):1-14.

[2] Bilinski A, Salomon JA, Giardina J, Ciaranello A, Fitzpatrick MC. Passing the test: a model-based analysis of safe school-reopening strategies. Annals of Internal Medicine. 2021.

[3] Kaiser AK, Kretschmer D, Leszczensky L. Social network-based cohorting to reduce the spread of SARS-CoV-2 in secondary schools: a simulation study in classrooms of four European countries. The Lancet Regional Health-Europe. 2021;8:100166.
