# Supplementary material for: Assessing the impact of lateral flow testing strategies on within-school SARS-CoV-2 transmission and absences: A modelling study
Source: PLoS Comput Biol. 2022 May 27;18(5):e1010158. doi: 10.1371/journal.pcbi.1010158 (PMC9182264; doi:10.1371/journal.pcbi.1010158)
Supplement: S7 Text — The impact of assuming that only a percentage of LFTs are taken correctly, thereby reducing overall test sensitivity, on the number of infections over the course of a half-term for strategies involving LFTs. (PDF) [file pcbi.1010158.s007.pdf]

Supporting Information. Assessing the impact of lateral flow testing strategies on within-school SARS-CoV-2 transmission and absences: a modelling study

Trystan Leng[1,2*], Edward M. Hill[1,2], Robin N. Thompson[1,2], Michael J. Tildesley[1,2], Matt J. Keeling[1,2], Louise Dyson[1,2]

1. The Zeeman Institute for Systems Biology & Infectious Disease Epidemiology Research, School of Life Sciences and Mathematics Institute, University of Warwick, Coventry, United Kingdom

2. JUNIPER – Joint UNIversities Pandemic and Epidemiological Research, https://maths.org/juniper/

S7 Text: The impact of LFT user error

The test probability profiles we used in our main analysis[1] were inferred from swab data taken from UK healthcare workers. While, to our knowledge, there have been no specific studies considering the extent of user error for LFTs, a content analysis of LFT supporting information documents found that human factors were often not sufficiently addressed within accompanying documents to mitigate improper user[2]. Accordingly, we consider the specific impact of user error when taking LFTs on the total number of infections over a half-term. User error in LFTs is implemented by scaling down the daily probability of testing positive to an LFT by a factor $\phi, 0 \leq \phi \leq 1$. For example, if a pupil would be expected to test positive to an LFT with probability p when 100% of tests are taken correctly, we assume that pupil tests positive to an LFT with probability ϕp when $100 \times \phi$% of tests are taken correctly.

As one would expect, the total number of infections over a half-term decreases as the percentage of LFTs taken correctly increases for all strategies involving LFTs (Figure A). A strategy of serial contact testing results in a higher number of infections than a strategy of isolating year groups irrespective of the proportion of LFTs taken correctly, while a strategy combining twice weekly mass testing with the isolation of year groups is always at least as good as the isolation of year groups alone. A strategy of twice weekly mass testing requires 85% of LFTs to be taken correctly to result in fewer infections than a strategy of isolating year groups, while a strategy combining twice weekly mass testing with serial contact testing requires 40% of LFTs to be taken to correctly result in fewer infections than the isolation of year groups strategy.

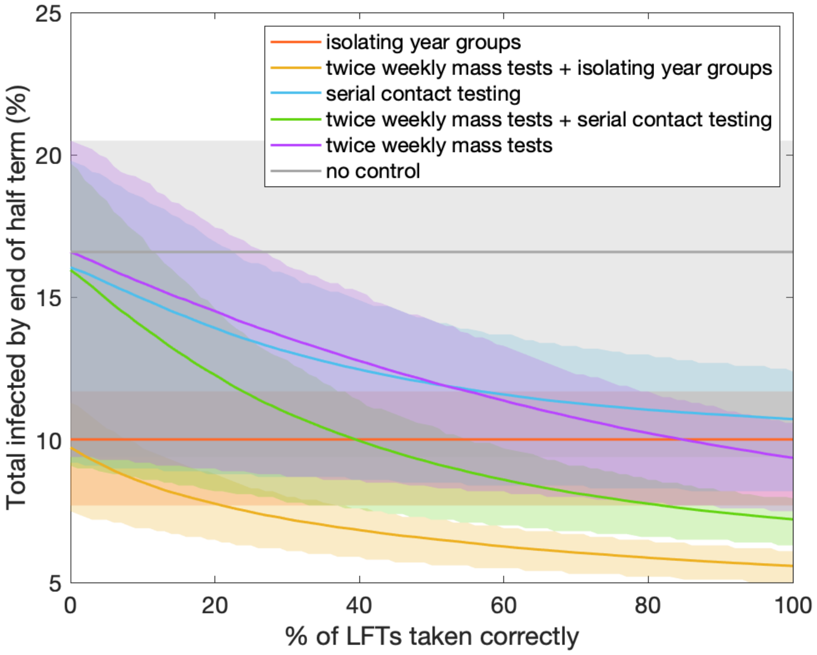

Figure A: **Exploring the impact of LFT user error on infections.** We study the impact of varying the percentage of LFTs taken correctly (from 0% to 100% in 1% increments) on the total number of pupils infected by the end of the half-term. Solid line traces correspond to the mean value attained from 2,000 simulations. Shaded envelopes represent the 50% prediction intervals (these regions contain 50% of all simulations at each timepoint). The strategies displayed are: no control (grey), twice weekly mass testing (purple), serial contact testing (blue), isolating year group bubbles strategy (orange), twice weekly mass testing (purple), combined twice weekly mass testing and serial contact testing (green), and combined twice weekly mass testing and isolation of year group bubbles (yellow).

References

[1] Hellewell J, Russell TW, Beale R, Kelly G, Houlihan C, Nastouli E, et al. Estimating the effectiveness of routine asymptomatic PCR testing at different frequencies for the detection of SARS-CoV-2 infections. BMC Medicine. 2021;19:106. Available from: https://bmcmedicine.biomedcentral.com/articles/10.1186/s12916-021-01982-x.

[2] Kierkegaard P, McLister A, Buckle P. Rapid point-of-care testing for COVID-19: quality of supportive information for lateral flow serology assays. BMJ open. 2021;11(3):e047163.
