# Supplementary material for: Assessing the impact of lateral flow testing strategies on within-school SARS-CoV-2 transmission and absences: A modelling study
Source: PLoS Comput Biol. 2022 May 27;18(5):e1010158. doi: 10.1371/journal.pcbi.1010158 (PMC9182264; doi:10.1371/journal.pcbi.1010158)
Supplement: Attachment — Submitted filename: Response to reviewers_ assessing.pdf [file pcbi.1010158.s008.pdf]

Response to reviewers: Assessing the impact of lateral flow testing strategies on within-school SARS-CoV-2 transmission and absences: a modelling study

We thank both reviewers for their valuable comments. We have addressed the comments made by both reviewers through expanded text in the discussion, the inclusion of an additional subplot in Figure 3, and a new subsection in the Supplementary information exploring the impact of user error when taking LFTs. We address the specific comments made by each reviewer in turn below.

Reviewers' comments are in blue text, our responses are in black text, while relevant sections of added text are included in italics. The specific part of a reviewer's comment that we are responding to is in bold blue font before the corresponding response. Sections of added text are in black italicised text, and the line numbers where they appear in the tracked changes versions of the manuscript are included.

Reviewer 1

This paper reports the results of a simulation study using an individual based model for the transmission of SARS-CoV2 within a school environment to compare and contrast the impact of alternative uses of rapid lateral flow tests in terms of the cumulative cases, absences and tests carried out.

The transmission model is necessarily largely assumptions based in the absence of useful empirical data at the school level to inform model development. Latent and infectious period distributions are based on the common set of empirical estimates from early in the pandemic used by most modelling studies.

1. Comment**:** The default configuration of the model assumes that transmission is well-mixed within year groups with limited exploration of alternative mixing patterns with assortative mixing between years and density versus frequency dependence (in supplementary information) and a range of different assumptions about prior immunity. **Given the well mixed assumption the individual based structure is a convenience (compared to compartmental population models) rather than a necessity but will also likely have some computational performance advantages**. **Likewise, given this assumption the size of the school years (and school population) is the only specific parameter distinguishes the model from (smaller) primary schools.**

   Response: While our model does not include heterogeneity in contact structure, the individual-based structure of our model is integral to our implementation of testing, as the time since an individual was infected influences the probability that individual will test positive. We now explicitly state that the only parameter distinguishing the model of secondary schools from primary schools is the size of school years. Our model focuses on the context of secondary schools specifically by considering testing strategies considered for secondary schools in England at various points of the

pandemic, and hence we believe the framing of our work in the context of secondary schools remains justified.

Added text (lines 444-446, main text): *"Our modelling approach could be adjusted to the primary school setting by altering the size and number of year groups to instead reflect the size and number of classes in a primary school."*

2. Comment: **The lack of an exploration of alternative transmission mechanisms is the largest limitation of the study. This is clearly described in the discussion, but I disagree with the justification (and necessity) for this assumption. We know from studies before the pandemic that social contact networks within schools are highly clustered - a network structure which is likely to limit and shape the rate of transmission within schools. The manuscript acknowledges this body of work but discounts the use of such data due to the likely changes in contact patterns due the pandemic. This is a valid point, however if anything social distancing measures might be expected to further fragment contacts and increase clustering. Network transmission scenarios (informed by empirical networks or simulated networks with similar patterns of clustering) would have provided a more robust evaluation of the sensitivity of results to model uncertainty and could very naturally be included within the chosen individual based modelling framework. Again, the time-critical nature of the policy relevance of such work makes such simplifying assumptions pragmatic (particularly given the lack of contemporary data) but it could be made clearer that such an exploration does not necessarily depend on carrying out time and resource consuming contact studies during a pandemic. The time-constraints under which work must be carried out to be relevant during a pandemic is a relevant and valid justification for the assumptions taken in itself and it would, in my view at least, be valuable to acknowledge it as such.**

   Response: We have now acknowledged in our discussion the time-constraints under which the work was undertaken as a reason for the simplifying assumptions surrounding contact structure within schools. We have also stated that the measures imposed may have impacted the level of clustering within schools. However, we believe that the impact of imposed control measures is not obvious. For example, contacts may be more structured around seating arrangements than friendship groups. If pupils have different seating arrangements in different classes, this may result in a less clustered contact network than one formed largely through friendship groups. In our main text, we have suggested that future studies incorporating aspects of network structure, and exploring the impact of levels of clustering on model outcomes, would be a valuable line of further research, and could be incorporated into our modelling framework.

   Amended text, added text in bold (lines 423-439, main text) : "*Fourth, as we consider a secondary school implementing a bubbling strategy at the level of year groups, we make the simplifying assumption of random mixing within year groups, ignoring the heterogeneity in contact structure within year groups. The omission of contact heterogeneity at the individual level is common in individual-based models of SARS-CoV-2 transmission in schools (Phillips et al. 2021, Bilinski et al. 2021, Asgary*

et al. 2021, Kunzmann et al. 2021), ***and was necessitated by the time-constraints under which this work had to be conducted within to be useful during a pandemic.*** *While studies prior to the COVID-19 pandemic have detailed contact patterns within schools (Salathe et al. 2010, Eames et al. 2011, Conlan et al. 2011), the implementation of stringent distancing measures within schools will have impacted such patterns.* ***Some studies have used contact patterns measured before the COVID-19 pandemic (Colosi et al, 2021), while*** *other studies have used structured expert judgement to inform contact patterns (Woodhouse et al. 2021) during the COVID-19 pandemic. However, data to robustly parameterise such patterns remains scarce,* ***and it may be expected that within-school distancing measures have impacted the fragmentation and clustering of school contact networks.*** *Going forward, contemporary surveys detailing contact patterns within schools and how these are affected by school-level distancing measures is an important line of research. Alongside these, modelling studies that assess the impact of heterogeneity* ***and clustering*** *in contact patterns on the effectiveness of school control strategies would be valuable.* ***The modelling approach outlined in this paper could be extended to investigate both aspects.****”*

3. Comment: While parameter uncertainty is not, and cannot be, systematically explored given the type of data available, sensitivity of the results to parameter assumptions is addressed through a one at a time (OAT) analysis. **This highlights the importance of the transmission parameters and test sensitivity which implies these parameters are likely to trade-off heavily against each other even if empirical data on transmission within schools was available.**

   Response: We thank the reviewer for highlighting the implication from our univariate sensitivity analysis. We have now included a comment in our sensitivity analysis section of the supplement that parameters may trade-off against one another, making their inference difficult even with empirical data on transmission.

   Added text (Supporting text S4, supplement): *“For all strategies, assuming that LFT or PCR sensitivity is higher than our baseline assumption reduces infections over the half-term, while assuming levels of within-school transmission, community transmission, or the relative infectiousness of asymptomatics are higher than our baseline assumption increases infections over the half-term. These parameters impact within-school transmission in opposing directions, meaning their inference may be challenging even with empirical data.”*

4. Comment: **The results are framed in terms of the trade-off between the number of infections averted, school absences and tests carried out. While the discussion acknowledges each of these have both a societal and economic costs the relative importance of each is not addressed which is vital for policy evaluation. Economic evaluations of public health control measures are routinely carried out using (albeit imperfect) measures such as DALYs. While I realise that there may not be reliable (or even any) quantitative estimates of these relative costs is this as they have not or cannot reasonably be evaluated. While the authors may well argue that this is a question of economics rather than biology, the policy implications of this work hinges on the answer whether**

this is a personal value judgement or something that can be more systematically quantified.

Response: We have now included a discussion of health economic approaches relevant to our modelling approach. By quantifying cases, absences, and tests, our approach makes only the first step in answering such questions. We acknowledge that a framework to quantify relevant factors is needed to evaluate optimal decisions from a health-economics perspective, while also acknowledging the difficulty in making such evaluations because of outcomes that are hard to directly measure.

Added text (lines 466-480, main text): *"By considering the level of absences realised and the volume of tests required under different reopening strategies, our study takes the first step in quantifying the indirect costs of SARS-CoV-2 transmission and control within schools. Future research should focus on incorporating the 'cost' of absences, tests, and cases amongst pupils into a health-economic framework, such as one that considers the impact of school control measures on quality adjusted life years (QALYs) (Verget et al. 2008). Health economic modelling has been used to complement epidemiological models for a range of diseases and contexts, helping to inform policy (Jit et al. 2008, Baguelin et al. 2010, Hill et al. 2020). However, a key challenge in implementing such an approach is quantifying the 'cost' associated with school days missed, how this varies across contexts, and to what extent this is mitigated by online learning. Further, other costs associated with school control strategies, such as the impact of control measures on the mental health of pupils, may be even harder to quantify. Studies prior to the COVID-19 pandemic have considered the impact of school days missed on attainment (Aucejo et al. 2016, Gottfried et al. 2017, Hancock et al. 2018) , while studies undertaken since the pandemic have attempted to quantify the impact of schools closures on loss of learning (Engzell et al. 2021) and mental health (Viner at al. 2022). Research synthesising existing knowledge into the harms associated with absences and control measures in the context of COVID-19, together with further research into these harms combined with epidemiological models of within-school transmission, is paramount to the design of optimal school control policies."*

5. Minor comment: **Introduction: While it is clearly a NPI, practicing good hand hygiene is not, unlike the other listed NPIs, really a social restriction and not as far as I know enforced legally in the same way. In the context of the point being made it's inclusion at the start of the list distracts a little from the main point being made.**
   Response: We have now omitted good hand hygiene from the discussion of NPIs.

To sum up, this is a well written account of a careful assumptions based modelling exercise to explore the trade-offs between different testing strategies in schools to limit the impact of SARS-CoV and associated control measures. The methods are clearly documented both within the paper and with additional detail in supplementary information. Full source code to replicate the analysis is linked and a reasonable attempt at exploring parametric and model sensitivity of the results has been carried out. Necessarily carried out under time constraints and used as part of the package of advice given to the UK government this work has already

demonstrated impact and it's publication is valuable in itself with respect to adding to the public record of the response to the ongoing pandemic.

Reviewer 2

Thank you for this interesting simulation study on different variations of repetitive testing in schools.

1. Comment: **The results would be more generalizable to other settings if a) it was not assumed that student participation was 100% in the main analysis (seems unlikely in most settings)**
   Response: While we acknowledge that 100% participation is an optimistic assumption, we wanted to explore the impact of such strategies when optimally implemented, particularly as we have no robust estimates of participation rates for the strategies considered. Accordingly, we have not amended Figures 1 and 2 to assume a lower level of participation. However, we have explicitly stated that the assumption of 100% participation is an optimistic one, and outline our justification for such, and we believe that through the results displayed in Figure 3 adequately address the impact of participation levels on the previous results.

   Amended text, added text in bold (lines 184-186, main text): "*Our main analysis assumed that all pupils participated in the school's control strategy.* ***We did so to explore the impact of such strategies when optimally implemented. However, in practice it is unlikely that all pupils would comply with a school's control strategy.*** *Accordingly, we also considered the impact of pupil participation in lateral flow testing.*"

2. Comment: **[The results would be more generalizable to other settings if] b) the error rates of the tests were adjusted to account for user error (both due to imperfect testing procedures, and possible changes due to changing variants).**

   Further comment: **Thank you for pointing out in the limitations that these results are actually quite optimistic. It would seem that "user error" in the testing with LFT is probably somewhat high in this population. Were any simulations performed assuming higher error rates?**

   Response: We thank the reviewer for suggesting the consideration of user error rates on the results of tests. In the supplement, we have now included a section and a figure addressing the impact of user error when taking LFTs on the success of strategies involving LFTs.

   Added text (lines 319-321, main text): *"If a large proportion of LFTs are taken incorrectly, reducing the sensitivity of such tests, a strategy of twice weekly mass testing combined with serial contact testing may become less effective at reducing infections than a strategy of isolating year group bubbles."*

   Further added text (Supporting text S9, supplement): *"The test probability profiles we used in our main analysis (Helewell et al. 2020) were inferred from swab data*

taken from UK healthcare workers. It may be expected that secondary school pupils are more likely to incorrectly administer a home test. While, to our knowledge, there have been no specific studies considering the extent of user error for LFTs, a content analysis of LFT supporting information documents found that human factors were often not sufficiently addressed within accompanying documents to mitigate improper user (Kierkegaard et al. 2021). Accordingly, we consider the specific impact of user error when taking LFTs on the total number of infections over a half-term. User error in LFTs is implemented by scaling down the daily probability of testing positive to an LFT by a factor φ, $0 \leq \varphi \leq 1$. *For example, if a pupil would be expected to test positive to an LFT with probability p when 100% of tests are taken correctly, we assume that pupil tests positive to an LFT with probability* φp *when* $100 \times \varphi$*% of tests are taken correctly.*

As one would expect, the total number of infections over a half-term decreases as the percentage of LFTs taken correctly increases for all strategies involving LFTs. A strategy of serial contact testing results in a higher number of infections than a strategy of isolating year groups irrespective of the proportion of LFTs taken correctly, while a strategy combining twice weekly mass testing with the isolation of year groups is always at least as good as the isolation of year groups alone. A strategy of twice weekly mass testing requires 85% of LFTs to be taken correctly to result in fewer infections than a strategy of isolating year groups, while a strategy combining twice weekly mass testing with serial contact testing requires 40% of LFTs to be taken correctly to result in fewer infections than the isolation of year groups strategy."

3. Comment: **In Figure 3, it would have been interested to see also the number of missed infections.**
   Response: We thank the reviewer for suggesting a subfigure of Figure 3 showing the number of missed infections. To address this, and to remain consistent with quantities displayed in Figures 1 and 2, we have now added a third subfigure to Figure 3, Figure 3(c), tracking the proportion of asymptomatic individuals identified.
